# Supplementary material for: Inflammatory factors and risk of lung adenocarcinoma: a Mendelian randomization study mediated by blood metabolites
Source: Front Endocrinol (Lausanne). 2024 Aug 27;15:1446863. doi: 10.3389/fendo.2024.1446863 (PMC11384989; doi:10.3389/fendo.2024.1446863)
Supplement: Supplementary file 1 [file DataSheet1.zip › supplementary files/S2/GCST90200247/forest.pdf]

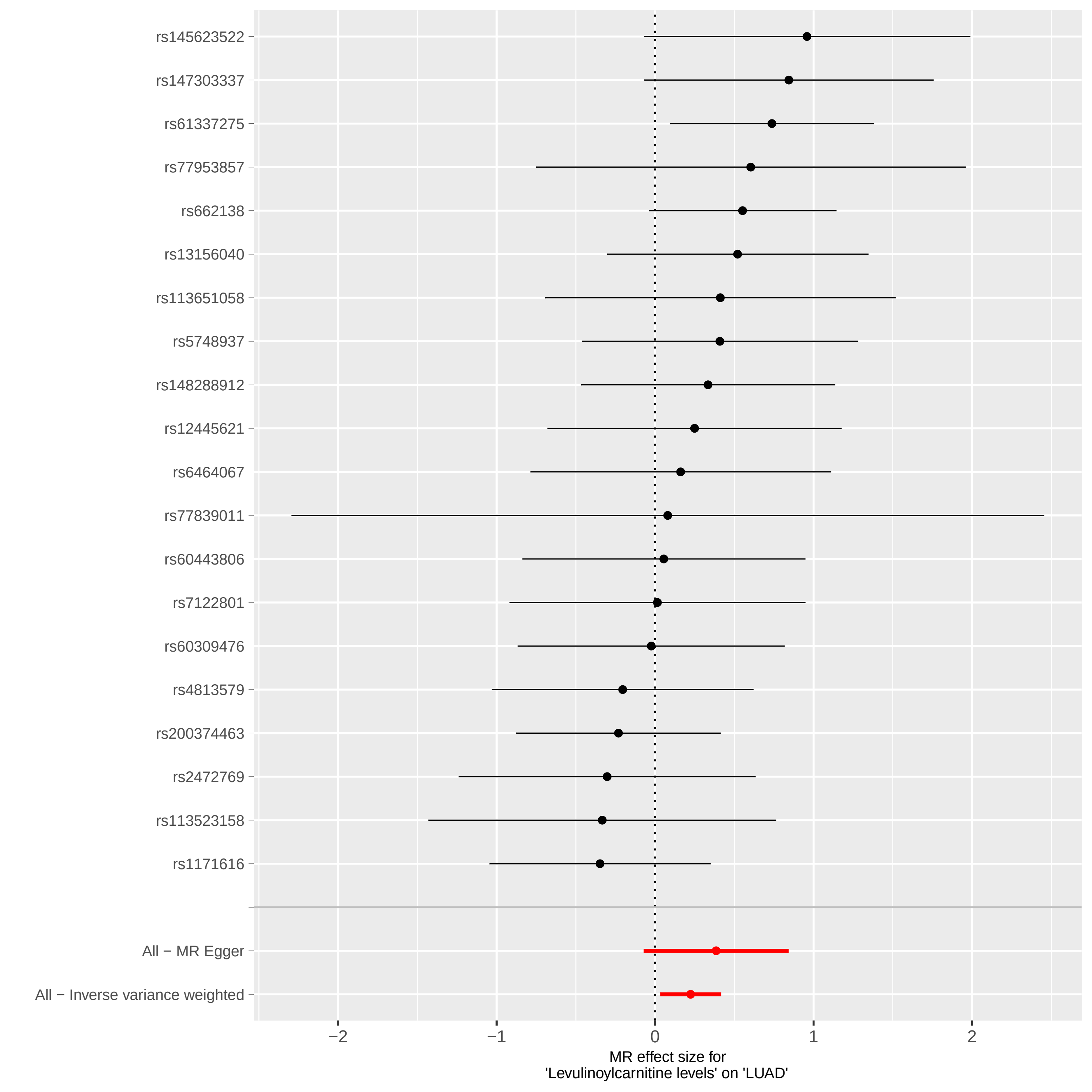
rs145623522
rs147303337
rs61337275
rs77953857
rs662138
rs13156040
rs113651058
rs5748937
rs148288912
rs12445621
rs6464067
rs77839011
rs60443806
rs7122801
rs60309476
rs4813579
rs200374463
rs2472769
rs113523158
rs1171616
All − MR Egger
All − Inverse variance weighted
−2
−1
0
1
2
MR effect size for
'Levulinoylcarnitine levels' on 'LUAD'
